# Air Pollution and Perinatal Mental Health: A Comprehensive Overview

**DOI:** 10.3390/jcm12093146

**Published:** 2023-04-27

**Authors:** Teresa Surace, Cecilia Quitadamo, Alice Caldiroli, Enrico Capuzzi, Fabrizia Colmegna, Guido Nosari, Elisa Borroni, Luca Fedrizzi, Valentina Bollati, Angela Cecilia Pesatori, Michele Carugno, Massimo Clerici, Massimiliano Buoli

**Affiliations:** 1 Department of Mental Health and Addiction, Fondazione IRCCS San Gerardo dei Tintori, Via G.B. Pergolesi 33, 20900 Monza, Italy; 2 Department of Medicine and Surgery, University of Milan Bicocca, Via Cadore 38, 20900 Monza, Italy; 3 Department of Neurosciences and Mental Health, Fondazione IRCCS Ca’ Granda Ospedale Maggiore Policlinico, Via F. Sforza 35, 20122 Milan, Italy; 4 EPIGET Lab, Department of Clinical Sciences and Community Health, University of Milan, 20122 Milan, Italy; 5 Epidemiology Unit, Fondazione IRCCS Ca’ Granda Ospedale Maggiore Policlinico, Via San Barnaba 8, 20122 Milan, Italy; 6 Department of Pathophysiology and Transplantation, University of Milan, 20122 Milan, Italy

**Keywords:** air pollution, perinatal mental health, affective disorders, psychotic disorders

## Abstract

Background: The aim of the present study was to summarise the available data about the link between air pollution exposure and the new-onset and severity of psychiatric disorders in pregnant women during the perinatal period. Materials and methods: We selected articles published until June 2022 on PubMed and the Web of Science. Pollutants included were PM_2.5_ (particulate matter 2.5 micrometres and smaller), PM_10_ (particulate matter 10 micrometres and smaller), NO_2_ (nitrogen dioxide), O_3_ (ozone), SO_2_ (sulphur dioxide), CO (carbon monoxide), PBDEs (polybrominated diphenyl ethers), PFAS (per- and polyfluoroalkyl substances), lead, and cadmium. The perinatal period was considered as the time of pregnancy until one year after childbirth. Results: Nine studies were included; most of them evaluated the association between exposure to air pollutants and the onset of Postpartum Depression (PPD). Two studies showed an association between, respectively, only PM_2.5_ and both PM_2.5_ and NO_2_ exposure and PPD onset 12 months after childbirth, while another study found a significant association between NO_2_ exposure and PPD occurrence 6 months after childbirth. PBDE blood levels were associated with more severe depressive symptoms. Lastly, one study observed a link between stressful symptoms and exposure to PM_2.5,_ PM_10_ during pregnancy. Conclusion: More comprehensive and uniform studies are required to make a roadmap for future interventions, given the growing relevance of issues such pollution and mental health, particularly during the perinatal period.

## 1. Introduction

The presence of mental well-being in the perinatal period is of fundamental importance both for women’s health and for the development of a beneficial mother-child bond [1]. On the other hand, women during pregnancy and postpartum are highly vulnerable to the development of psychiatric disorders [2]. One of the most difficult areas for public health concerns perinatal mental illnesses; if not properly managed, these conditions have significant detrimental consequences not only for women, but also for family members and the relationship between mothers and newborns [1,3]. For these reasons, paediatric providers are currently invited to monitor not only the health of the child but also that of the parents and caregivers [4]. It is estimated that during the perinatal period, about 12% of women suffer from depression [5], and even a higher percentage (till 30%) reports clinically significant anxiety symptoms [6]. In addition, a first postpartum psychotic disorder happens in 0.25–0.6 cases per 1000 births [7].

Different factors contribute to the vulnerability to psychiatric disorders in the perinatal period, including biological, psychosocial, and environmental features [8]. With regard to biology, epigenetic modifications [9,10], as well as increased inflammation and low vitamin D plasma levels [11], appear to contribute to vulnerability to depression during pregnancy and postpartum. On the other hand, ongoing conflict with partners, poor social support, adverse life events [8], and unemployment [12] were reported as predictors of poor mental health in the perinatal period.

Air pollution represents one of the most important causes of premature death worldwide [13]. It is currently ranked among the top five risks for attributable deaths globally [14,15], increasing the vulnerability to a number of medical conditions such as respiratory and cardiovascular diseases (e.g., hypertension), along with other chronic disorders such as type 2 diabetes, chronic kidney disease, obesity, autoimmune disease, and dementia [16]. With regard to this latter diagnosis, most studies were not centred on a specific type of dementia (only two reports focused, respectively, on Alzheimer’s disease and vascular dementia) [17]. In this framework, poor air quality has been recently identified as a potential contributor to mental health worsening [18]. A recent meta-analysis highlighted that all main air pollutants and particularly short- and long-term exposure to particulate matter ≤ 2.5 μm (PM_2.5_) might increase the risk of depression [19].

The underlying biological mechanisms explaining the negative effect of air pollution on mental health include increased inflammation with activation of microglia in the Central Nervous System (CNS) [20] and epigenetic modifications such as those regarding “clock genes” [21]. Of note, clock genes regulate circadian rhythms, including those regarding basic life functions such as sleep or appetite [22]. In particular, transcriptional/translational feedback mechanisms involving CLOCK-BMAL, a protein dimer complex, have been identified as the main regulators of circadian rhythms [23,24]. Recent studies have highlighted that epigenetic modifications in clock and other genes (different degrees of methylation) can contribute to the development of depression and anxiety disorders during the perinatal period [25], as a result of hypomethylation of clock genes *CRY1* and *CRY2* [9] and modulation of *HERV-W*, the latter implicated in maternal immune tolerance during pregnancy [26]. Women during the perinatal period are particularly vulnerable to the negative effects of air pollution as it was demonstrated, even in physiological pregnancy, that a delay of circadian rhythms, particularly in the third trimester, prepare breastfeeding [27]. Furthermore, increased inflammation due to air pollution would modify the regulation of oxytocin, a neuropeptide that plays an important role in newborn-mother attachment and breastfeeding [28]. In addition, this neuropeptide demonstrated anti-inflammatory properties, thus preventing the negative effects on biological systems by depressive disorders [27,28]. Of note, the maternal neuroendocrine system, which is critical for normal homeostasis and allostatic activation, undergoes dramatic changes during pregnancy, mainly due to the effects of the developing placenta [29,30]. The disruption of physiological hypotalamic-pituitary-adrenal (HPA) axis activity during pregnancy through stress-induced elevation in maternal cortisol levels [30] contributes to immune system dysregulation. Particularly, the hypotalamic corticotropic releasing factor (CRF) [31] and glucocorticoids entering the brain [32] trigger microglia and neuronal changes. Oxytocin plays a key role in inhibiting this process, attenuating HPA-stress response with paracrine mechanisms [28]. Of note, air pollution can promote systemic inflammation with a modulation of clock gene expression, in turn influencing glucocorticoid receptor transcription [33]. Furthermore, increased systemic inflammation due to air pollution can activate and influence the HPA-axis functioning [34]. Finally, it is worth mentioning that pregnant women have an increased ventilation rate due to higher oxygen demand and lower oxygen-binding capability together with high metabolic requests from the brain. All of these factors contribute to susceptibility to air pollution since pollutants affect the respiratory system and are able to cross the blood–brain barrier and trigger neuroinflammation, as mentioned above [35,36,37].

In light of the increasing evidence of a negative effect of air pollution on mental health and the importance of psychological wellbeing during pregnancy and postpartum, purpose of the present overview is to critically summarize the available data about the association between poor quality of air and psychiatric diagnoses, such as mood, anxiety, or psychotic disorders, in women during the perinatal period.

## 2. Materials and Methods

This review was performed according to the Preferred Reporting Items for Systematic Reviews and Meta-Analyses (PRISMA) guidelines [38]. A search was performed in the following psychiatric databases, PubMed (National Library of Medicine Bethesda, Maryland) and the Web of Science, to identify relevant papers.

Moreover, the registries of US NIH (National Institutes of Health) clinical trials were consulted. All the original articles written in English from 1987 to 31 May 2022, with available abstract and full texts, were included.

Two authors subsequently checked and extracted data from included articles: paper author and title, publication year, characteristics of the study (design, sample size, duration of study, type of pollutant, diagnosis, and psychometric tools). If relevant data were not reported in the selected articles, the corresponding author was contacted to obtain further information.

The search was performed using the keywords: “pollution” AND (“postpartum” OR “peripartum” OR “perinatal”) AND (“depression” OR “anxiety” OR “bipolar” OR “psychotic”). Inclusion criteria were: (1) original articles; (2) mean age of patients over 18 years; (3) reported information about mental health during peripartum period; (4) topic of the article focused on the association between air pollution and mental health in the peripartum period, defined as pregnancy and one year after delivery [39].

Exclusion criteria were: (1) reviews, meta-analyses, commentaries, letters, case reports, pooled analyses, comments, case studies, study protocols; (2) studies conducted on animals; (3) studies about the relation of mental health with air pollution outside the perinatal period; (4) studies about the relation of pollen with mental disorders; (5) studies about the exposition to insecticides and fertilisers; (6) data on individuals largely or totally overlapping to samples whose results had been already published (7) articles not written in English language. The search strategy and the inclusion and exclusion criteria followed PRISMA guidelines [40].

Quality rating was performed according to criteria by Armijo-Olivo et al. [41] and the effect sizes were calculated for all the primary results as Cohen’s d when possible. The protocol of this study was registered on PROSPERO (CRD42022336618).

## 3. Results

### 3.1. Literature Retrieval and Study Characteristics

**Figure 1:**
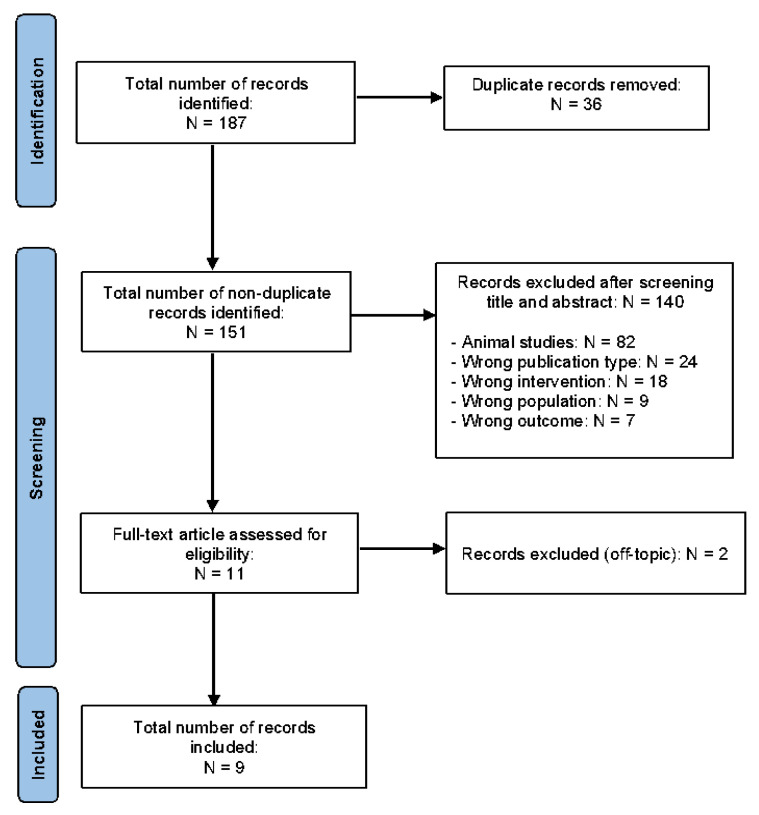
Prisma diagram.

A total number of 187 articles was screened. A total of 36 papers were duplicates and 142 were excluded for above mentioned criteria. Nine studies finally satisfied the inclusion criteria (Figure 1).

The main characteristics of included studies are shown in Table 1. Seven articles were prospective studies [42,43,44,45,46,47,48] and two were retrospective ones [49,50]. Four studies were conducted in the USA, the other five respectively in China, Republic of Korea, Taiwan, Mexico, and Turkey.

Seven studies evaluated the effect of exposure to the following air pollutants on women’s perinatal mental health: PM_2.5_, PM_10_, nitrogen dioxide (NO_2_), ozone (O_3_), sulfur dioxide (SO_2_) and carbon monoxide (CO). Vuong and collaborators focused on polybrominated diphenyl ethers (PBDEs) and per- and polyfluoroalkyl substances (PFAS) [44]: these compounds are anthropogenic chemical substances dispersed in the air from different stuff including car interiors, firefighting foams, and textiles [51,52,53,54]. These substances have received growing interest in recent years due to the potential association between exposure to these compounds and the onset and severity of various medical conditions including Coronavirus [55] and dysfunction of thyroid hormones [56].

Finally, one study [42] analysed the effect of lead and cadmium exposure on mothers’ perinatal mental health: these elements usually contaminate water and soil, but they can be found also in aerosol particles [57,58].

Seven of nine studies evaluated the effect of pollutant exposure on the severity of depressive symptoms assessed by different self-rated psychometric scales: Postnatal Depression Scale (EPDS) (4 studies) [42,43,48,49], Center for Epidemiologic Studies-Depression (CES-D) scale (one study) [45], Beck Depression Inventory-II (BDI-II) (one study) [44], 36-Item Short Form Survey (one study) [47]. One research [50] selected cases of post-partum depression (PPD) based on SNOMED (Systemized Nomenclature of Medicine) codes. One study [46] evaluated the effect of air pollution on prenatal maternal stress assessed by the Perceived Stress Scale (PSS).

### 3.2. Air Pollution Exposure and Maternal Depression

Duan and co-authors reported that exposure to PM_10_, CO and NO_2_ during the whole pregnancy and exposure to SO2 during the second trimester were associated with an increased risk of developing PPD at 6 weeks after delivery [48]. Another study found that PPD occurrence 6 months after childbirth was related to NO_2_ exposure during early pregnancy (first trimester) but not to PM_2.5_ and CO [47]. Other authors reported an association between NO_2_ exposure during the entire pregnancy and PM_2.5_/NO_2_ exposure during mid-pregnancy (second trimester) with maternal depression onset 12 months after childbirth [45]. Further research [43] elucidated that increased PM_2.5_ exposure in mid-pregnancy (second trimester) was associated with more severe depressive and anhedonia symptoms after delivery, particularly in Black women. A retrospective study found that a 5 μg/m^3^ change in mean PM_2.5_ exposure during pregnancy was associated with an increased risk of PPD 6 months after delivery [49]. In contrast, Zhang and co-authors [50] reported that women experiencing PPD were more likely to reside in neighborhoods with lower air pollutant concentrations. Similarly, no significant associations were found regarding lead and cadmium levels in breast milk and PPD diagnosis [42]. Blood levels of some PBDEs and PFAs during pregnancy (BDE-4 and perfluorooctanoate-PFOA, perfluorooctane sulfonate-PFOS) were found to contribute to severity of depressive symptoms after delivery (BDE-4 and PFOA 4 weeks after childbirth; PFOS both in the short-and in the long-term after delivery) [44].

### 3.3. Air Pollution and Maternal Stress

One study reported that the maternal susceptibility to stressful events at the end of pregnancy is modulated by changes in exposure to PM_2.5_ and PM_10_ during the whole pregnancy and to O_3_ in the third trimester. This susceptibility resulted more evident for women with a lower level of education. The association between PSS scores and PM_10_ was stronger in the spring season and no associations were found concerning NO_2_ exposure [46].

### 3.4. Air Pollution and Other Mental Disorders

No results were found concerning the onset of anxiety, psychotic and bipolar disorders in relation to air pollutants during the perinatal period.

## 4. Discussion

Despite the heterogeneity of designs, the results of the included papers show small/medium effect of the exposition of PM_2.5_ during pregnancy on the risk of developing clinically significant depressive symptoms during the postpartum (3 positive medium quality studies for a total of 1246 women with a Cohen’s d effect size ranging from 0.24 to 0.26 versus 1 weak quality negative study) [43,45,47,49]. In addition, preliminary findings from one study would indicate an effect of this pollutant in increasing the susceptibility to stressful events at the end of pregnancy [46]. NO_2_ is the other pollutant as well as PM_2.5_ that seems to increase the vulnerability to developing depressive symptoms in case of exposition during pregnancy (3 positive studies for a total of 31,577 women with a Cohen’s d effect size ranging from 0.27 to 0.39 if the exposition to whole pregnancy is considered) [38,45,47]. Positive and preliminary findings also regard PM_10_ and SO_2_ [47], while available data are contradictory for O_3_ [45] and CO [47,48]. The negative effect of PBDEs and PFAs on women’s perinatal mental health should be further clarified [44].

As mentioned above, the effect of O_3_ is controversial with one study associating the concentrations of this pollutant with the level of perceived stress during the third trimester of pregnancy [46] and another that reported that lower concentrations of O_3_ increased the risk of PPD [50]. This is not surprising because O_3_ concentrations and toxicity are dependent on the temperature and play a prominent role during summer or heat waves [59].

The negative effects of air pollutants on mothers’ perinatal mental health, in particular of PM_2.5_ and NO_2_, can be motivated by different biological reasons. First, these toxics can contribute to increased systemic inflammation: NO_2_ exerts strong oxidizing effects [60], while PM_2.5_ induces DNA damage [61]. Chronic over-inflammation has a negative effect on the Central Nervous System (CNS) for the passing of different cytokines through the blood brain barrier and the consequent dysregulation of neurotransmission [62]. The excessive inflammation in the CNS induces the activation of the indoleamine 2,3-dioxygenase that accelerates the degradation of tryptophan from which serotonin is synthesized [63]. Second, PM_2.5_ is able to pass through the blood brain barrier exerting a direct toxic effect in the CNS. Finally chronic peripheral damage (e.g., of respiratory system) can induce changes in bone marrow of the skull thus sustaining inflammation in the brain with neurotoxic effects [64]. Moreover, short- and long-term exposure to PM was found to be associated with increased plasma levels of cytokines such as IL-6, IL-1𝛽, and TNF𝛼 [65,66]. As mentioned in the introduction, the effects of neuroinflammation during the perinatal period are particularly detrimental for the role of neuropeptides and circadian rhythms for the physiological functioning of women in this specific phase of life [25].

On the other hand, different authors demonstrated a dysregulation of the immune system with prominent inflammation in women affected by perinatal depression [26,67]. In addition, the presence of affective disorders during pregnancy was associated with a number of unfavourable obstetric outcomes including low birth weight or preterm birth [68,69]. All these observations make it essential to intervene on all modifiable factors that can reduce the risk of psychiatric conditions during the perinatal period including air pollution [37]. The prevention should therefore especially protect pregnant women, particularly those with a biological predisposition to suffer from mood disorders, from residing in places with high concentrations of air pollutants [70].

## 5. Conclusions

In conclusion, although some promising data support the impact of air pollution on mental health of women during the perinatal period, various considerations must be carried out about the accuracy of the findings presented in this overview.

First of all, we considered the perinatal period until one year after delivery, but air pollutants could have a negative effect on longer periods [44] after childbirth. In this framework, the entanglement between air pollution and different factors affecting biological systems in the long-term should be taken into account [34]. Of note, potential triggers of late-onset PPD are represented by breastfeeding or the recurrence of menstrual cycles, which could lead to hormonal changes, affecting mood [71]. Furthermore, recent studies show that the exposure to air pollution during pregnancy is associated with a higher risk of neurodevelopmental disorders in the offspring, thus representing a further stressful factor for new mothers [72].

Second, some of the included studies did not take into account environmental factors potentially affecting the risk of mood disorders such as season, climate, hours of light [73,74].

Third, the included studies were conducted in geographical areas with different degrees of air pollution: for example, Asian countries are well-known for showing average air pollution levels that are higher than those found in Western countries [75].

Finally, it is also important to underline the fact that the results presented in this study do not demonstrate a causality between air pollution and poor mental health in the perinatal period. Many factors (not controlled in studies on this topic) may have influenced the correlation between air pollution and women’s perinatal mental health including psychosocial factors (e.g., economic poverty, global migration) [76] or biological aspects (greater biological susceptibility to stressful factors in this particular period of women’s life) [77]. Future studies with a rigorous methodology are needed to confirm the preliminary positive associations between air pollution and poor perinatal mental health.

## Figures and Tables

**Table 1:** Summary of methods and main results of the included studies.

Study (Country)	Design	Quality (EPHPP)	Study Participants	Psychometric Tools	Psychiatric Disorders	Perinatal Window	Pollutants	Source of Exposure Assessment	Adjustment Variables	Effect Size (Cohen’s d)	Main Results
Duan et al., 2022 [48] (China)	Multi-city prospective cohort study	Moderate	10,209 pregnant women in 5 hospitals from Shangai, Hangzhou and Shaoxing (October 2019–February 2021)	EPDS (cut-off score: 10 and 13) at 6 weeks postpartum	PPD	Pregnancy–6 weeks postpartum	PM_2.5_ PM_10_ SO_2_ CO NO_2_ O_3_	Local ambient monitoring stations	Socio-demographic variables, obstetric variables, season, city, daily temperature	Entire pregnancy: PM_10_: 0.21 (for a 10 μg/m^3^ increase) CO: 0.46 (for a 0.1 μg/m^3^ increase) NO_2_: 0.27 (for a 10 μg/m^3^ increase) 2nd trimester: SO_2_: 0.05 (for 1 mg/m^3^ increase)	Exposure to PM_10_, CO and NO_2_ during the whole pregnancy is associated with an increased risk of developing depression at 6 weeks postpartum SO_2_ exposure during the second trimester increases the risk of PPD
Bastain et al., 2021 [45] (USA)	Prospective cohort study	Moderate	180 women from the MADRES project cohort in Los Angeles, California (2015–2020)	CES-D scale (cut-off score: 16) at 12 months postpartum	Maternal depression	Pregnancy–12 months postpartum	PM_2.5_ PM_10_ NO_2_ O_3_	Local ambient monitoring stations	Socio-demographic variables, history of depression, air conditioning use, average temperature, study recruitment site	Entire pregnancy: NO_2_: 0.39 2nd trimester: PM_2.5_: 0.24 NO_2_: 0.53	Exposure to NO_2_ during the whole pregnancy is associated with an increased risk of developing depression at 12 months postpartum Exposure to NO_2_ and PM_2.5_ during the second trimester is associated with an increased risk of developing depression at 12 months postpartum
Lamichhane et al., 2021 [46] (Republic of Korea)	Prospective cohort study	Moderate	2153 pregnant women followed up in different medical centers in the Seoul metropolitan area (2007–2015)	PSS scale assessed at the 36th week of pregnancy (third trimester)	Prenatal maternal stress (increase in PSS scores)	Pregnancy	PM_2.5_ PM_10_ NO_2_ O_3_	LUR models	Socio-demographic variables, obstetric characteristics, medical comorbidities, maternal smoking, alcohol during pregnancy	Entire pregnancy: PM_2.5_: 0.93 PM_10_: 1.32 3rd trimester: O_3_: 0.75	During the whole pregnancy IQR increases in exposure to PM_2.5_ and PM_10_ were associated with 0.37- and 0.54-point increases in PSS scores During the third trimester IQR increases in exposure to O_3_ were associated with 0.30-point increases in PSS scores
Shih et al., 2021 [47] (Taiwan)	Prospective cohort study	Weak	21,188 mother-infant pairs from Taiwan Birth Cohort Study-TBCS (2005)	36-Item Short Form Survey administered 6 months after childbirth	PPD	Pregnancy–6 months postpartum	PM_2.5_ CO NO_2_	Hybrid Kringing-LUR and LUR-based machine learning models	Socio-demographic variables, obstetrical variables, breastfeeding, infant general health status, perinatal smoking or smoking history, perinatal alcohol consumption, ambient temperature	1st trimester: NO_2_: 0.01 per IQR increases in exposure (10.67 ppb)	PPD occurrence was significantly related to exposure to NO_2_ during first trimester of pregnancy (early pregnancy)
Zhang et al., 2021 [50] (USA)	Retrospective observational study	Weak	EHR data on 8949 pregnant women from an urban academic medical center in New York City (2015–2017)	PPD diagnosis within 1 year after childbirth based on SNOMED codes	PPD	Pregnancy–12 months postpartum	PM_2.5_O_3_	LUR models	Socio-demographic variables, clinical problems, medication prescriptions, built environment, prenatal care variation, pregnancy characteristics and outcomes	NA	Women who experienced a prenatal care pattern with highest rates of PPD were more likely to reside in neighbourhoods with lower air pollutant concentration
Niedzwiecki et al., 2020 [49] (Mexico)	Retrospective cohort study	Moderate	509 mothers from the PROGRESS study in Mexico City (July 2007–February 2011)	EPDS (cut-off score: 13) administered during pregnancy, at 1 and 6 months postpartum	PPD	Pregnancy–6 months postpartum	PM_2.5_	LUR models	Socio-demographic variables, negative life events during pregnancy, environmental tobacco smoke, birth season	0.26	A 5 μg/m^3^ change in PM_2.5_ average exposure during pregnancy was associated with increased PPD risk at 6 months
Vuong et al., 2020 [44] (USA)	Prospective cohort study	Moderate	377 women from the HOME study conducted in Cincinnati, Ohio (March 2003–February 2006)	BDI-II at 20-week gestation and 7 times postpartum (4 weeks, 1,2,3,4,5 and 8 years)	Maternal Depression	Pregnancy–12 months postpartum	PBDEs (BDE-28, -47, -99, -100, -153 and ƩPBDEs) PFAS (PFOA, PFOS, PFHxS, PFNA)	PBDEs and PFAS blood levels at 16 ± 3 weeks of gestation were collected, then chromatography and mass spectrometry analysis were performed	Socio-demographic variables, self-reported marijuana use during pregnancy, serum cotinine (tobacco use or environmental smoke exposure), serum ƩPCBS, maternal IQ	Estimated score differences in BDI scores at 4 weeks after delivery by 10-fold increases in serum PBDE concentrations (ng/g lipid) during pregnancy: BDE-28: 0.65 (NS) BDE-4: 0.23 BDE-99: 0.80 (inverse association) BDE-100: 0.12 (inverse association) BDE-153: 0.49 ∑PBDEs: 0.41 (inverse association) Estimated score differences in BDI scores at 4 weeks after delivery by 1-ln unit increases in serum PFAS concentrations(ng/mL) during pregnancy: PFOA: 1.69 PFOS: 1.56 PFHxS: 0.59 (NS) PFNA: 1.34 (NS) Estimated score differences in BDI scores at 1 year after delivery by 10-fold increases in serum PBDE concentrations (ng/g lipid) during pregnancy: BDE-28: 2.80 (NS) BDE-47: 2.61 (NS) BDE-99: 2.07 (NS) BDE-100: 2.93 (NS) BDE-153: 2.44 (NS) ∑PBDEs: 2.68 (NS) Estimated score differences in BDI scores at 1 year after delivery by 1-ln unit increases in serum PFAS concentrations(ng/mL) during pregnancy: PFOA: 0.85 (NS) PFOS: 0.78 PFHxS: 0.39 (NS) PFNA: 0.41 (inverse association-NS)	PBDEs and PFAs blood levels during pregnancy were found to contribute to severity of depressive symptoms after delivery
Sheffield et al., 2018 [43] (USA)	Prospective cohort study	Moderate	557 mothers who delivered at ≥ 37 weeks of gestation from the ACCESS project cohort (2002–2007)	EPDS (cut-off score: 13) at 6 and 12 months postpartum	PPD	Pregnancy–12 months postpartum	PM_2.5_	Data from U.S. Environmental Protection Agency (EPA)	Socio-demographic variables, prenatal smoking, season of delivery	NA	Increased PM_2.5_ exposure in mid-pregnancy (second trimester) was associated with severity of depressive and anhedonia symptoms, particularly in Black women
Örun et al., 2011 [42] (Turkey)	Prospective cohort study	Moderate	144 mothers residing in a suburban area who delivered in Ankara (July-September 2006)	EPDS scale (cut-off score: 13)	PPD	2 months postpartum	Pb Cd	Pb and Cd levels in breast milk at 2 months postpartum were determined by ICP-MS	Maternal and infant characteristics	Pb: 0.11 Cd: 0.10	No correlation was found between breast milk Pb and Cd levels and EPDS scores

Legend: ACCESS—asthma coalition on community, environment, and social stress; BDI-II—beck depression inventory; Cd—cadmium; CES-D—center for epidemiologic studies depression scale; CO—carbon monoxide; EPHPP—effective public health practice project; HER—electronic health records; EPDS—edinburgh postnatal depression scale; ICP-MS—inductively coupled plasma mass spectometry; IQR—interquartile range; LUR—land use regression; RR—risk ratio; NA—not applicable; NS—not statistically significant; NO_2_—nitric dioxide; O_3_—ozone; Pb—lead; PBDEs—polybrominated diphenyl ethers; PFAS—poly- and perfluoroalkyl substances; PFHxS—perfluorohexane sulfonate; PFNA—perfluorononanoate; PFOA—perfluorooctanoate; PFOS—perfluorooctane sulfonate; PM_2.5_—particulate matter with an aerodynamic diameter less than or equal to 2.5 μm; PM_10_—particulate matter with an aerodynamic diameter less than or equal to 10 μm; PPD—postpartum depression; Ppb—1 part per billion; PSS—perceived stress scale; SNOMED—systemized nomenclature of medicine; SO_2_—sulphur dioxide.

## Data Availability

The datasets generated during and/or analyzed during the current study are available from the corresponding author on reasonable request.
